# Impact of pregnancy on inborn errors of metabolism

**DOI:** 10.1007/s11154-018-9455-2

**Published:** 2018-09-10

**Authors:** Gisela Wilcox

**Affiliations:** 1 School of Medical Sciences, Faculty of Biology Medicine & Health, University of Manchester, Manchester, UK; 2 The Mark Holland Metabolic Unit, Salford Royal Foundation NHS Trust, Salford, Greater Manchester, M6 8HD UK

**Keywords:** Pregnancy, Inborn errors of metabolism, Metabolic disorders, Mitochondrial, Urea cycle, Fatty acid oxidation

## Abstract

Once based mainly in paediatrics, inborn errors of metabolism (IEM), or inherited metabolic disorders (IMD) represent a growing adult medicine specialty. Individually rare these conditions have currently, a collective estimated prevalence of >1:800. Diagnosis has improved through expanded newborn screening programs, identification of potentially affected family members and greater awareness of symptomatic presentations in adolescence and in adulthood. Better survival and reduced mortality from previously lethal and debilitating conditions means greater numbers transition to adulthood. Pregnancy, once contraindicated for many, may represent a challenging but successful outcome. Successful pregnancies are now reported in a wide range of IEM. Significant challenges remain, given the biological stresses of pregnancy, parturition and the puerperium. Known diagnoses allow preventive and pre-emptive management. Unrecognized metabolic disorders especially, remain a preventable cause of maternal and neonatal mortality and morbidity. Increased awareness of these conditions amongst all clinicians is essential to expedite diagnosis and manage appropriately. This review aims to describe normal adaptations to pregnancy and discuss how various types of IEM may be affected. Relevant translational research and clinical experience will be reviewed with practical management aspects cited. Based on current literature, the impact of maternal IEM on mother and/or foetus, as well as how foetal IEM may affect the mother, will be considered. Insights gained from these rare disorders to more common conditions will be explored. Gaps in the literature, unanswered questions and steps to enhance further knowledge and systematically capture experience, such as establishment of an IEM-pregnancy registry, will be summarized.

*Dedicated to my mother Patricia Lorraine (Young) Wilcox 1928 – 2017 who taught me my first lesson in Rare Diseases: "Is it rare? Or is it just rarely diagnosed?"*

## Introduction

Once mainly in the domain of paediatrics, inborn errors of metabolism (IEM), or inherited metabolic disorders (IMD) the preferred term in clinical practice, represent a growing specialty in adult medicine [1]. Reasons for this include improved diagnosis through expanded newborn screening programs, identification of potentially affected family members and greater awareness of symptomatic presentations in adolescence and in adulthood. Greatly improved survival and reduced mortality from previously lethal and debilitating conditions have enabled survival into adulthood and greater participation in society [2]. Sigmund Freud described the stage of adulthood as being ‘to love and to work’ [3]. Pregnancy in women with IEM can be seen, as a successful outcome of survival and transition into adulthood, achieving as normal a life as possible.

Once thought to be contraindicated for many conditions, there are now accumulating reports of successful pregnancies in a wide range of IEM [4]. There do remain significant challenges however, given the biological stresses of pregnancy, parturition and the puerperium. Outcomes are most favourable where the diagnosis of an IEM is known, allowing preventive and pre-emptive management. Unrecognized metabolic disorders have been, and remain, a preventable cause of maternal and neonatal mortality and morbidity. Increased awareness of these conditions amongst all clinicians is essential to expedite diagnosis and manage appropriately. Helpful and detailed recent reviews have been published on the clinical experience [4] including practical management of such pregnancies [5]. Improved understanding may enable anticipation of possible problems especially where current knowledge and experience is limited and thinking from first principles may be required.

The focus of this review is to describe normal adaptations to pregnancy, discuss how various types of IEM may be affected in this setting and integrate current experience of such pregnancies in the literature. Where relevant, insights gained from these rare IEM to more common conditions will be explored.

Finally, gaps in the literature, unanswered questions and steps to enhance further knowledge and experience will be summarized.

## Biological adaptations to pregnancy

To understand the potential impact pregnancy may have in women with IEM we must first consider general adaptations to pregnancy. Anatomical and systemic physiological changes, from conception to the puerperium, are, necessarily effected by modification of subcellular metabolism under genomic and endocrine influence.

We will therefore discuss in turn, anatomical and body composition changes from conception to the post-partum period, physiological and metabolic adaptations to pregnancy.

### Anatomical changes

**Fig. 1:**
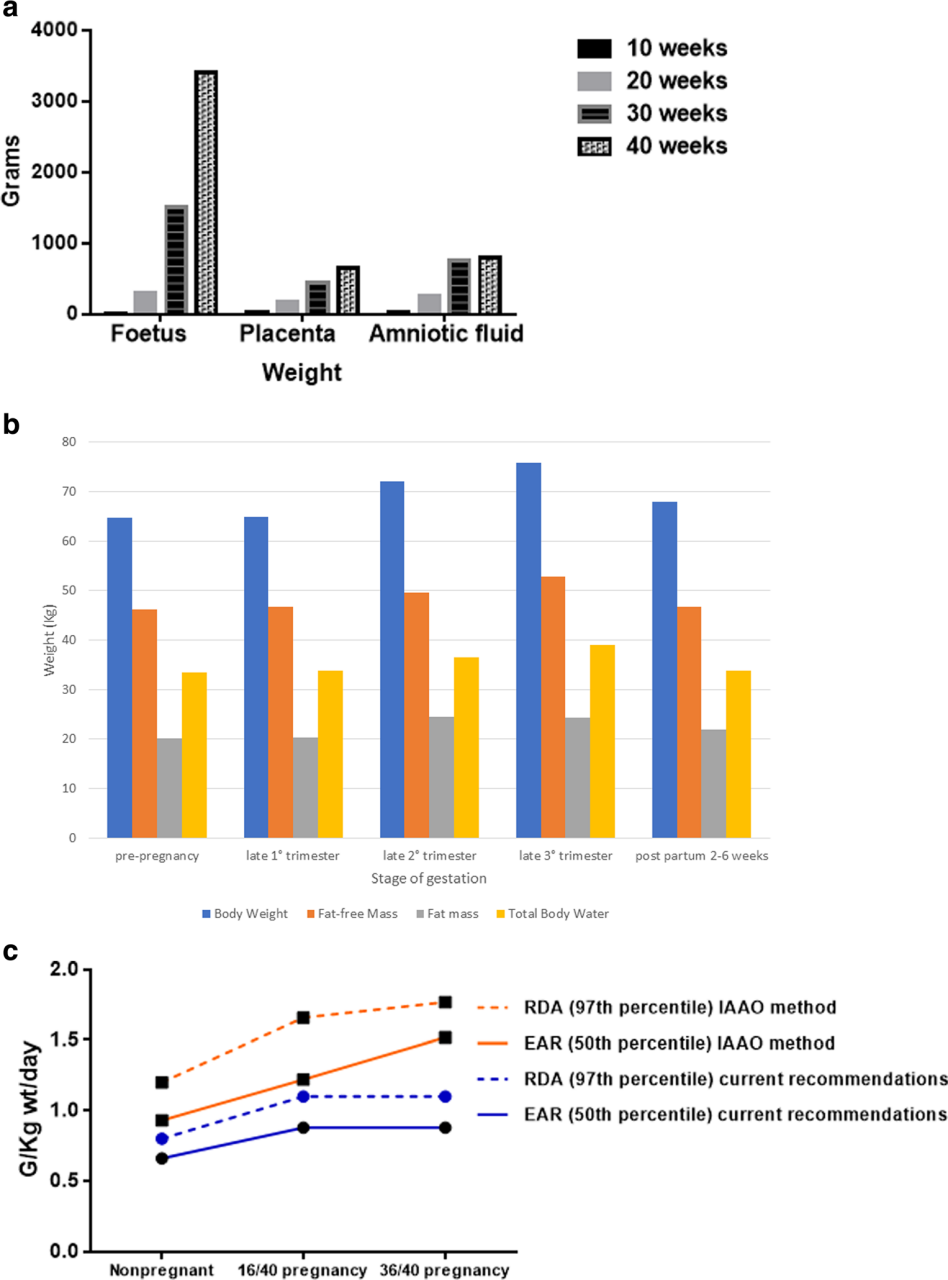
**a Foeto-placental growth across pregnancy.** Adapted from King JC, Reference [7]: Physiology of pregnancy and nutrient metabolism. Am J Clin Nutr. 2000;71(5 Suppl):1218s–25s. **b Maternal body composition changes across pregnancy and the puerperium.** Adapted from *Kopp-Hoolihan* et al in Reference [6]: Widen EM, Gallagher D. Body composition changes in pregnancy: measurement, predictors and outcomes. Eur J Clin Nutr. 2014;68(6):643–52. **c Protein requirements in pregnancy by method.** Recent methodology by *in vivo* amino acid oxidation (IAAO) suggests protein requirements, whether by Estimated Average Requirement (50th percentile) or Recommended Daily Allowance (97th percentile) are significantly higher than previous estimates. Source: adapted from content in (reference [8]): Elango R, Ball RO. Protein and Amino Acid Requirements during Pregnancy. Adv Nutr. 2016;7(4):839s44s. https://academic.oup.com/advances/article/7/4/839S/4568693

Over the 40 gestational weeks of pregnancy through the post-partum period there are substantial changes, beyond foetal development, in maternal body composition [6] Fig. 1a and b). Current knowledge of body composition in pregnancy, as well as relevant methodology, has been reviewed [6].

These changes are reflected in the requirements for protein and its component amino acids, essential fatty acids, calcium, iron and other key micronutrients, with energy storage and utilization during pregnancy being derived from carbohydrate (glucose) and lipids [7] *(see section* *2.3**).*

### Physiological adaptations

Alterations in total body water and its compartments contribute to cardiovascular changes observed during pregnancy [9]. Increased plasma volume, hence venous return, affects preload. Systemic vascular resistance, reduced via oestrogen and nitric oxide mediated vasodilatation, decreases afterload. S*troke volume*, the blood volume pumped into the systemic circulation per cardiac cycle, increases by 20–30%. Maternal heart rate increases from the first to the third trimester. Therefore, cardiac output increases by 30–50% during a singleton pregnancy. This supports uterine and placental blood flow, accounting for around 25% of the total. Increased renal, mammary and skin blood flow support the increased oxygen requirements of these maternal organs [9]. The substantially increased metabolic demands during pregnancy are discussed in section 2.3.

Respiratory physiological changes include tidal volume increasing by 30–50%, respiratory rate rising by 1–2 breaths/min and development of a mild respiratory alkalosis. Oxygen consumption increases by 30%, with maternal metabolic rate increasing by 15% [9]. Systemic and renal vasodilatation increase renal blood flow. Glomerular filtration rate increases 40–50% by the end of the first trimester with creatinine clearance increasing correspondingly. This contributes to altered renal threshold for glucose reabsorption. Glomerular membrane charge selectivity alters, which together with raised GFR, increases urinary albumin and protein excretion [9]. Changes in maternal bone and mineral metabolism occur during pregnancy and post-partum. The foetus accretes 30 g calcium, 20 g phosphorus and 0.8 g magnesium by end gestation. This was recently reviewed [10]. Altered gut motility with reduced lower oesophageal sphincter tone, delayed gastric emptying and slowed intestinal transit time are well described, attributed largely to progesterone [9].

Normal liver function includes synthetic, excretory and metabolic functions. Synthesis of proteins includes clotting proteins, lipids and transport proteins like albumin. Excretory functions encompass catabolism and excretion of bile acids, detoxification and biotransformation of xenobiotics and hormones. As the sentinel organ of intermediary metabolism, metabolic functions include nitrogen metabolism, transamination and ureagenesis; regulation of carbohydrate metabolism includes galactose metabolism, glycolysis, glycogenolysis and glycogen storage, as well as lipid synthesis, storage and metabolism [11].

Pregnancy significantly influences liver function. Synthesis of proteins, such as lipoproteins and coagulation factors, increase under hormonal influence [9]. A hypercoagulable state results, particularly relevant in homocystinuria where thrombosis risk is already increased [5]. Effects on intermediary metabolism are discussed further below.

### Metabolic adaptations to the demands of pregnancy

#### Energy balance and calorie requirements in pregnancy

The energy required to support the metabolic demands of an average full-term singleton pregnancy is approximately 38,000 Kcal with the major energy cost coming near term with estimated BMR increase of around 230 Kcal per day [7].

In the first two trimesters of pregnancy, maternal anabolism predominates, with enhanced insulin sensitivity. Where food availability permits, maternal fat- and fat-free mass increase over this period [6, 7]. From around 30 weeks’ gestation, placental hormones and adipocytokines drive increasing insulin resistance [12, 13], favouring maternal catabolism, liberating glucose, amino acids and lipids to support exponential foetal growth. Maternal catabolism may be exaggerated in the third trimester where nutritional state is borderline [14].

#### Carbohydrate metabolism

Lipolysis-derived glycerol becomes a preferred substrate for maternal gluconeogenesis [14] while maternal glucose is diverted for foetal consumption. In late pregnancy, around 50% of maternal glucose production is utilized by the foetus. Fasting maternal glucose levels are reduced throughout pregnancy reflecting enhanced hepatic and placental uptake. In later pregnancy peripheral (muscle) insulin resistance is associated with higher postprandial glucose levels. Glucose passes across the placenta via GLUT1 facilitated diffusion, down a concentration gradient [13]. These processes are reviewed in greater depth in recent reviews of metabolism in gestational diabetes [13].

#### Amino acid metabolism

**Fig. 2:**
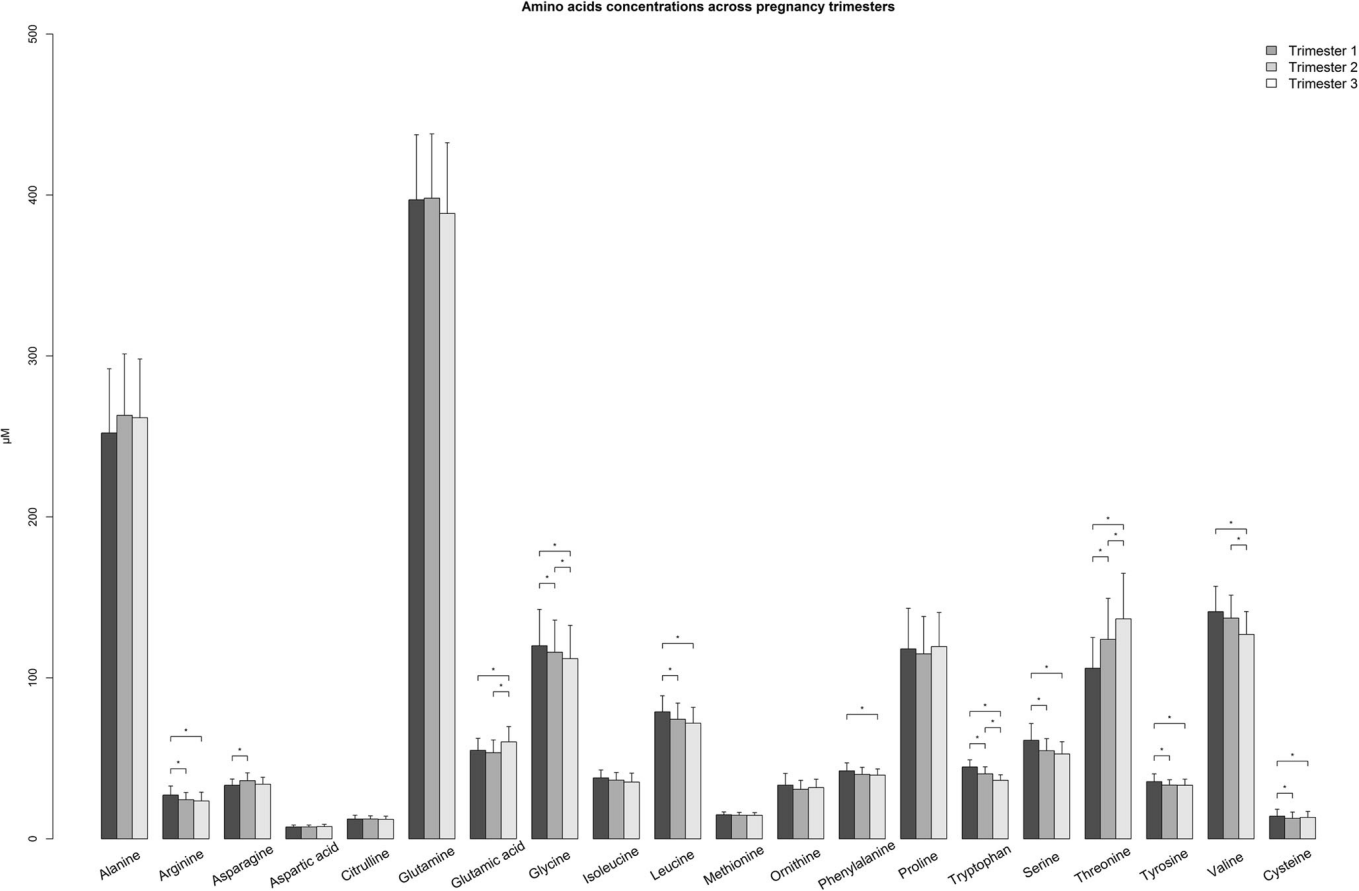
**Amino acid concentrations across pregnancy trimesters.** Barplot comparing plasma amino acid concentrations across trimesters among pregnant women. Median (+ Interquartile range/2) was plotted. * *p*-value <0.00017, p-value was calculated by Mann-Whitney U Test between trimesters. Source: (Reference [17]) Lindsay KL, Hellmuth C, Uhl O, Buss C, Wadhwa PD, Koletzko B, et al. Longitudinal Metabolomic Profiling of Amino Acids and Lipids across Healthy Pregnancy. PLoS One. 2015;10(12):e0145794

Amino acids are actively and differentially transported across the placenta [15, 16] supporting foetal growth, while maternal plasma amino acid levels are progressively lowered [8, 17] Fig. 2. These changes are evidenced by recent metabolomic studies [18]. The differential between maternal and foetal plasma amino acid levels varies between individual amino acids and across trimesters. This has implications for inherited inborn errors of protein metabolism, such as phenylketonuria, in pregnancy, where foetal uptake is greater relative to some other amino acids.

Protein requirements are increased from early pregnancy and increase throughout gestation [8, 19]. Contemporary methods such as *in vivo* amino acid oxidation suggest previous estimates of gestational protein requirements, based historically from extrapolation of nitrogen balance studies in men, may be insufficient, especially in late pregnancy, Fig. 1c [8].

#### Lipid metabolism

Maternal plasma VLDL cholesterol and triglycerides increase during pregnancy, with oestrogen-mediated decreased hepatic lipoprotein lipase [20]. HDL and LDL are additionally triglyceride enriched via cholesterol ester transport protein (CETP). [12, 14]. Placental hormone-sensitive lipase releases free fatty acids, including long chain essential fatty acids, to support foetal growth and development. Long chain fatty acids (LCFA) are transported across the plasma membrane via the LCFA transporter, then esterified and bound to fatty acid binding proteins (FABP). Carnitine, via the plasma membrane carnitine transporter, enters the cytoplasm and combines with the LCFA esters, and is itself transported across the inner and outer mitochondrial membranes by Carnitine Palmitoyl Transporters (CPT) 1 and 2 respectively. Medium chain (MCFA) and short chain fatty acids (SCFA) can enter the cell and into the mitochondria independently of carnitine and its transporters [21].

Human placental tissue is mitochondrial-rich [22, 23] and generates ATP from beta oxidation of very long chain fatty acids (VLFA), cleaving them into shorter subunits, as well as utilizing medium chain fatty acids (MCFA) and short chain fatty acids (SCFA) for ATP synthesis, [24]. Peroxisomal functions are complex, include elongation of EPA to DHA, apparently enhanced in the foeto-placental unit, as well as metabolism of more complex lipids e.g. phytanic acid a branched long chain fatty acid of ruminant origin [25–27].

Ketones generated from mitochondrial fatty acid oxidation are used as a maternal fasting energy supply, as well as for foetal energy and brain development [13]. Cholesterol is essential for foetal development and metabolism. There is evidence for both de-novo foetal synthesis as well as placental transport of maternal cholesterol [13].

The importance of lipid metabolism in human, as opposed to rat, pregnancy is now much better recognized. One can speculate on the significant interspecies differences in relative brain size and development [28].

The clinical importance of lipid metabolism in normal pregnancy is highlighted by the complex foeto-maternal pathology that occurs in pregnancies where there are inborn errors of lipid metabolism, in the mother and/or foetus [29].

### Parturition

The physiology of labour is complex and has recently been reviewed from the perspective of myometrial function [30]. From a metabolic standpoint it represents prolonged and intense muscular activity where the energy requirements are substantial [30]. Whether altered cellular metabolism e.g. acidosis in specific IEM influences myometrial function in labour has not been directly studied.

### Post-partum

Following delivery of the foetus and placenta, maternal endocrine and metabolic status changes abruptly, while the newborn rapidly adjusts to post-uterine life. Maternal catabolism is pronounced with energy and nutrients being diverted to milk production from the third postpartum day. Uterine involution is especially rapid in the first 10–14 days post-partum; by six weeks post-partum it has returned to pre-pregnancy size. This mobilizes amino acids for lactation. The abrupt post-partum oestrogen decline similarly facilitates maternal skeletal calcium mobilization [10] Post-partum maternal body composition changes resulting from catabolism are evident in Fig. 1b.

This is a therefore a period of exceptionally high risk for decompensation of inborn metabolic disorders in at risk-mothers and neonates. Many such disorders present for the first time in the post-partum or neonatally.

In this section of the article we have reviewed the anatomical, physiological and metabolic adaptations that occur in normal pregnancy and puerperium as a framework to review what happens when an IEM is present in the mother and/or in specific situations, the foetus.

## Types of IEM and how they may be impacted upon by pregnancy

IEM represent numerous individually rare conditions with current collective prevalence of >1:800 [31]. They can be variously classified. As broad categories they encompass respectively, disorders of intermediary metabolism, mitochondrial energy metabolism and organelle-based disorders such lysosomal storage disorders [32]. Effects of pregnancy may differ between these groups which will therefore be discussed separately, with emphasis on disorders of intermediary and of mitochondrial energy metabolism.

In exploring the types of impact pregnancy may have on IEM, insights from translational research and clinical experience will be reviewed. Where relevant this will include effects of pregnancy on maternal IEM, maternal IEM on the foetus and/or foetal IEM on maternal pathophysiology.

These first two categories are exemplified by well described inborn errors of protein and amino acid metabolism.

### IEM of intermediary metabolism

#### Inborn errors of protein and amino acid metabolism

##### Urea cycle disorders

**Fig. 3:**
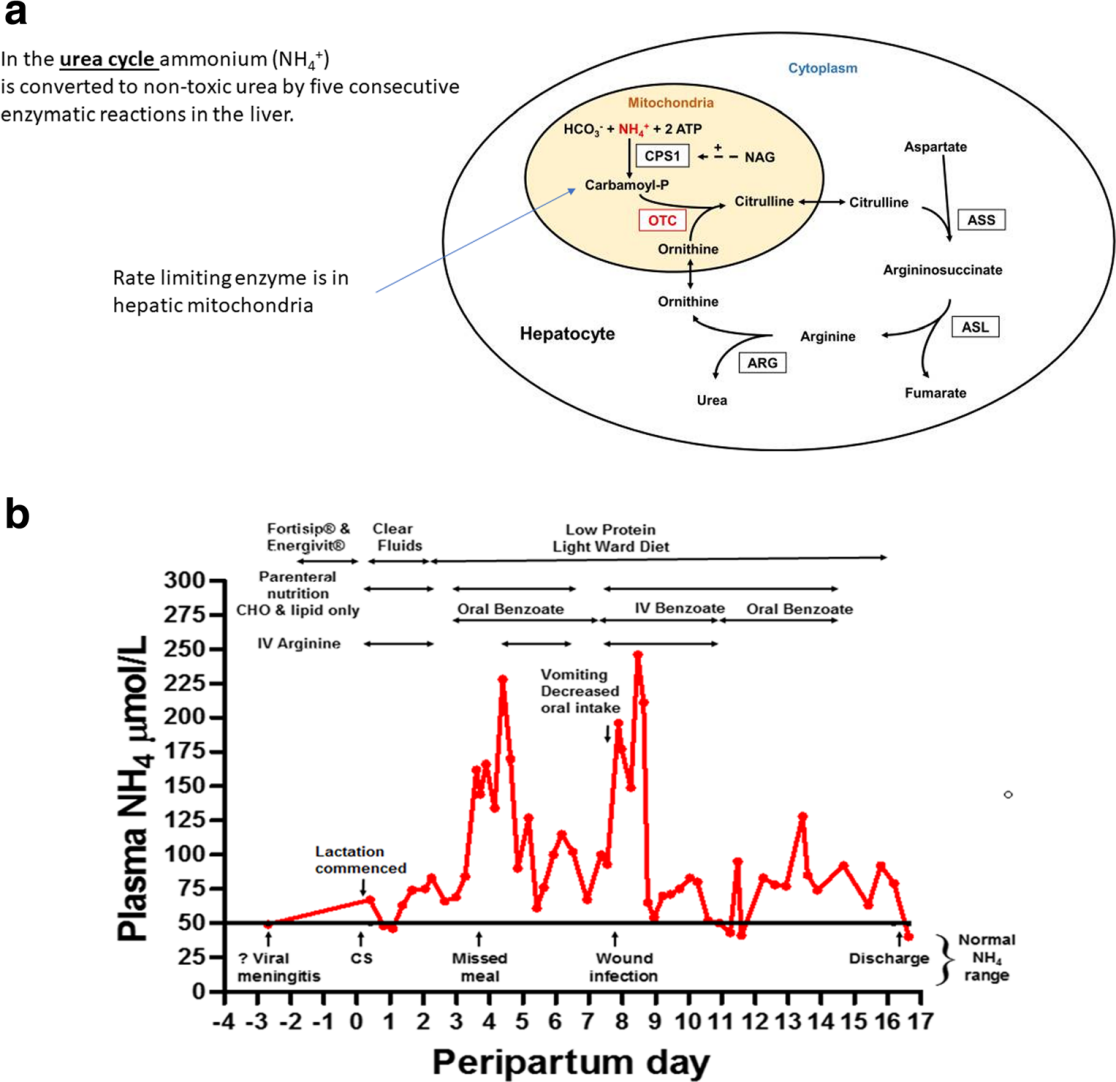
**a The urea cycle.** In hepatocytes, the rate-limiting, ATP-dependent enzyme carbamoyl phosphate synthetase 1 (CPS1), which is allosterically activated by N-acetyl glutamate (NAG), produced by N-acetyl glutamate synthase (NAGS), *not shown*, and ornithine transcarbamylase (OTC) are located in the mitochondria; argininosuccinate synthetase (ASS), argininosuccinate lyase (ASL) and arginase (ARG) are in the cytoplasm. Inherited defects in any of these enzymes can cause recurrent episodes of hyperammonemia. Defects in two mitochondrial transporters, *not shown*, may also result in hyperammonemia. Source: Adapted from reference [33]: Laemmle A, Gallagher RC, Keogh A, Stricker T, Gautschi M, Nuoffer JM, et al. Frequency and Pathophysiology of Acute Liver Failure in Ornithine Transcarbamylase Deficiency (OTCD). PLoS One. 2016;11(4):e0153358. **b Post-partum course of term OTC pregnancy.** Complicated post-partum course in a female with OTC due to partial X chromosome deletion. Abbreviations: CHO – carbohydrate; CS – Caesarian Section; NH_4_ – ammonia; OTC - ornithine transcarbamylase; IV – intravenous. Source: Goldstein R, Smith N, Strauss BJG & Wilcox G. Protein aversion and disordered eating in OTC deficiency: a challenge for pregnancy and post-partum management in a female heterozygote. 25th DMIMD, London, UK April 2011

The urea cycle, first described by Hans Krebs in 1932, is a series of 6 enzymes located in the liver. Its major role is to convert ammonia generated from excess protein intake or catabolism into urea. The urea cycle also regulates acid-base balance, consuming bicarbonate, and generates the amino acids citrulline, arginine and ornithine. Urea cycle dysfunction may occur where activity of any of the six enzymes, or their required transporters, co-factors or energy (as ATP) is lacking. The first three urea cycle enzymes are intra-mitochondrial while the remaining enzymes are cytosolic, Fig. 3a [34].

Inheritance of ornithine transcarbamylase (OTC) is X-linked, with more variable phenotypic expression in ‘carrier’ females; the remaining enzymes are autosomal recessive. Deficiencies of any of these enzymes may be associated with hyperammonaemia, [32] though is less frequent in arginase deficiency [35].

*Toxic effects of ammonia on the brain* have been well described [36] and may manifest clinically as neuro-psychiatric symptoms such as agitation, mood swings, perseveration, delirium, hallucinations, seizures, ataxia, psychomotor retardation and fluctuating conscious state as well as coma. Sodium valproate, though contraindicated in pregnancy, is well known to unmask inherited UCD due to its inhibition of urea cycle enzymes [37] It is important to note that adults with genetic causes of hyperammonaemia tolerate lower plasma levels than those commonly seen in chronic liver disease and are less tolerant than neonates and children with IEM [36].

*Gastrointestinal symptoms* of UCD may include aversion to dietary protein, anorexia, nausea, cyclical eating patterns, and acute liver failure (ALF) associated with hyperammonaemia, recently reviewed by Bigot [33]. Beyond paediatric presentations, Laemmle et al. reported ALF in 40% of confirmed OTC symptomatic females, including adults [38]. Ammonia-induced suppression of hepatic protein synthesis, mitochondrial dysfunction, damage and cell death appear to be the mechanisms involved, based on various lines of evidence, including liver biopsy [38]. The late rise in transaminases relative to ammonia elevation is a clue to the aetiology. Intramitochondrial UCD may be more prone to ALF and energy deficit may add to mitochondrial compromise. *Importantly, the liver failure is reversible with timely and appropriate treatment of hyperammonaemia, avoiding the need for liver transplantation.*

*Triggers for acute metabolic decompensation* are typically excess exogenous protein load and/or the presence of catabolic stressors such as infection, surgery, glucocorticoid therapy and inadequate caloric intake. Prevention is by maintaining a protein intake closely matched to requirements to minimize protein catabolism, and, ensuring adequate intake of non-protein calories. Ammonia scavenging medications enhance nitrogen excretion via alternative pathways and may be used preventively as well as during acute decompensations. A sick-day regimen, with ample non-protein calories to meet daily energy requirements is used during intercurrent illness to minimize catabolism. Intravenous glucose (and/or lipid) and hospitalization may be needed if enteral administration is not possible. Management of metabolic decompensation requires monitoring of ammonia, plasma amino acids, acid base, routine bloods and neurological observations, as well investigation for the underlying cause and any potential complications. It should be noted that clinical status can change dramatically within a few hours. In severe hyperammonaemia, haemofiltration may be needed. For further details, see references [32, 39].

##### Urea cycle disorders in pregnancy

In the face of increased metabolic demands, and requirements for protein, amino acids and energy there is a risk of decompensation where these are not met and/or catabolism is excessive overwhelming already limited capacity for ureagenesis and ammonia consumption.

Decompensation is riskiest where the diagnosis is unrecognized and/or treatment is delayed or not given. Conversely, better outcomes are observed where the UCD (and pregnancy) diagnosis is known.

In 1990 a seminal New England Journal of Medicine case series [40], reported post-partum coma and death in previously undiagnosed OTC-deficient female carriers. Subsequent numerous publications described various pregnancy outcomes in OTC [4, 41–55] and other urea cycle disorders: citrullinaemia type 1 [56–61], ASA/ASL deficiency [62–64], CPS deficiency [65] and lysinuric protein intolerance [66–68]. Currently, no reports of pregnancy outcome in either NAGS deficiency, Arginase deficiency, Citrin deficiency (citrullinaemia type 2) or Hyperornithinaemia-Hyperammonaemia-Homocitrullinaemia (HHH) syndrome are published.

*Adverse pregnancy outcomes reported have been principally neurological, psychiatric or hepatic.* The importance of acute hepatic failure as a manifestation of metabolic decompensation has been not been fully appreciated until recently [38]. Such presentations, occurring at any stage in pregnancy, may have gone undiagnosed. Whether they are represented in referrals to liver transplant units is worthy of investigation. Mitochondrial dysfunction [69] and liver disease will be discussed further with fatty acid oxidation disorders in pregnancy.

*Complications of hyperammonaemia in pregnancy can masquerade as more common problems.* Nausea, vomiting, headaches, mood disturbance and seizures may be attributed to hormonal changes. Mental status change post-partum has been diagnosed as post-partum psychosis or depression [53, 58, 59, 70]. Hyper-ammonaemic liver failure initially attributed to fatty liver of pregnancy, was considered unusual presenting in *early* pregnancy with hyperemesis, weight loss and prominent depression of synthetic function, [56, 57, 60, 71]. Hyperemesis gravidarum (HG) a risk factor for metabolic decompensation, due to caloric deficit, may be both a cause, and consequence, of hyperammonaemia. Late first trimester pregnancy weight loss from hyperemesis, malnutrition and institution of parenteral nutrition in an undiagnosed OTC heterozygote has caused fatal hyperammonaemic encephalopathy [46]. Glucocorticoids recommended for HG [72], an intercurrent condition [56] or anticipated pre-term delivery [42], may aggravate a catabolic state.

*Understanding the metabolic adaptations to pregnancy provides a framework for understanding and anticipating the impact on an IEM.*

*Most reported complications occur in early pregnancy and post-partum.* Progressive foetal and maternal second trimester anabolism generally confers greater metabolic stability. However, we have seen second trimester decompensation in OTC deficiency (G Wilcox unpublished observations), manifest by psychiatric disturbance, and responsive to intravenous arginine infusion; deficiency of this conditionally essential amino acid likely coincided with increased protein requirements.

Third trimester protein tolerance is generally greater due to increasing protein requirements, but, failing adequate intake^1^ and/or catabolic stressors [42] metabolic decompensation may occur. This is consistent with accelerated maternal catabolism in late pregnancy.

Peripartum multidisciplinary planning in known patients includes clinical observation, ammonia monitoring and avoidance of prolonged fasting, using protein-free nutrition orally, or if necessary, parenterally.

*Most hyperammonaemic decompensations have been reported post-partum.* Uterine involution takes 6 weeks but occurs rapidly in the first two weeks. The strength of catabolic drive from days 3–11 is such, that metabolic instability may still occur despite proactive appropriate management [4] Table 1. Additional catabolic stress may result from caesarian section, birth trauma, infection e.g. wound infection, mastitis (Fig. 3b). Blood transfusion may represent an added protein load. Breast feeding is possible so long as caloric intake is adequate. Table 1 summarizes largely previously unpublished experience from an historical case series.

**Table 1:** Historical case series of pregnancies in women with urea cycle disorders

Case	Age (years)	Diagnosis	Parity at delivery	Past pregnancy complications	Symptoms pre-pregnancy	Metabolic complications of pregnancy	Post-partum ammonia rise (*N* < 50 μmol/l)	Nutritional deficiencies	Breast feeding
1	40	OTC	2	‘Psychosis’ day 3 post-partum	Protein aversion	1° Trimester coma; Ammonia 288	117 day 5	–	Yes
2a^a^	33	OTC	1	Elective termination for affected foetus	Protein aversion decompensation	Post-partum hyperammonaemia	226 day 4 246 day 8	zinc selenium vitamin B12, iron protein	Yes
2b	35	OTC	2	As above	Protein aversion decompensation	Post-partum & 3° Trimester hyperammonaemia	105 day 2 150 day 4 125 day 9	zinc selenium vitamin A vitamin B12 protein essential fatty acids	Yes
3a	32	OTC	3	No	Protein aversion	–	–	vitamin D, vitamin B12, iron magnesium	Yes
3b	35	OTC	4	As above	Protein aversion	Gestational diabetes	–	As above	Yes
4a	37	OTC	1	N/A	Protein aversion	Mild post-partum hyperammonaemia	105 day 3–4 107 day 9	vitamin D, vitamin B12 iron	Yes
4b	39	OTC	2	As above	Protein aversion	–	–	As above	Yes
5	25	OTC	1	N/A	Protein aversion anxiety depression	2° & 3° Trimester altered mental status very low arginine normal ammonia	–	vitamin D iron	Yes
6^b,c^	19	Citrullinaemia	1	Past miscarriage	Nil	1–2° Trimester hyperemesis, ↓8Kg, weight loss, acute liver failure & hyperammonaemia: NH3 165	–	vitamin D	Yes

^a^Goldstein R, Smith N, Strauss BJG & Wilcox G. Protein aversion and disordered eating in OTC deficiency: a challenge for pregnancy and post-partum management in a female heterozygote. 11th DMIMD, London, UK April 2011

^b^McCarthy EA Wilcox G, Paulsen G, Walker SP. Mid-trimester severe liver failure as an adult presentation of an inborn error of urea cycle metabolism. Royal Australian and New Zealand College of Obstetricians and Gynaecologists Annual Scientific Meeting, Sydney, September 2013

^c^Reference number [55]

***Phenylketonuria*** (PKU) – *an example where the main impact is on the developing foetus.*

Phenylketonuria, is well known, with widespread neonatal screening pioneered by Robert Guthrie and others, since the 1960s [73].

PKU is due to absent or dysfunctional phenylalanine hydroxylase, which converts phenylalanine to tyrosine. Untreated, it leads to severe mental retardation and marked mood and behavioural disturbances [73]. Excess phenylalanine is toxic to the developing brain and completes with other large neutral amino acids e.g. tryptophan crossing the blood brain barrier. Together with deficient tyrosine, this causes marked neurotransmitter derangement, with deficiencies of dopamine, noradrenaline and serotonin [74]. Excess phenylalanine increases oxidant stress [75], impairs cholesterol synthesis [75] and activates osteoclasts [76].

PKU outcome was revolutionized by Horst Bickel’s therapeutic diet, still used today. Extreme natural-protein restriction, supplemented with micronutrient-fortified phenylalanine-free amino acid-based supplements to meet nutritional requirements was instituted neonatally [74]. Close blood-spot monitoring of phenylalanine levels, maintained throughout development, has enabled attainment of near-potential IQ [73]. Adherence to such dietary stringency is difficult for many; alternative therapies e.g. tetrahydrobiopterin (BH4) or Kuvan®, co-factor for PAH, may be limited to those with residual enzyme activity and/or access [74, 77–79]. Many adults, including women of childbearing age, are lost to follow-up, often due historically, to ceasing in adolescence or earlier [73, 74] This is concerning as excess maternal blood phenylalanine is highly teratogenic throughout gestation. Active placental phenylalanine transport further elevates foetal blood levels [16].

##### Maternal PKU syndrome

In 1956, Charles Dent reported adverse neurological sequelae of maternal PKU in non-PKU children, suggesting toxicity of phenylalanine on foetal brain development [80]. Prevention by gestational dietary phenylalanine restriction advised in Mabry’s landmark 1963 paper [80], was trialled from mid-gestation in 1968, and preconception from 1979, while isolated case reports of microcephaly, intellectual disability, growth retardation and cardiac defects were published [81].

The full maternal PKU syndrome report in 1980, including biochemical threshold, reviewed published and unpublished data from all metabolic centres [81, 82]. This pivotal study reported offspring small for gestation age (SGA) in 40%, microcephaly in 73%, congenital heart disease in 12% and 92% were intellectually disabled [82]. Recent meta-analysis by Prick 2012 confirmed these findings with lower prevalence likely due to *inclusion* of women with milder genotypes, manifest as hyperphenylalaninemia (HPA) and representing about 50% of the total PKU / HPA population, previously excluded from Lenke & Levy’s original analysis. SGA was seen in in 19.2%, microcephaly in 46.2%, congenital heart disease in 6.6% and 46.9% were intellectually disabled. Facial dysmorphism was reported in 50% (odds ratio 4.0), the first trimester being the critical risk-period [83].

This underscores the importance of systematic data collection for rare disease registries, with pregnancy outcome *including the long-term follow-up of children born to mothers with IEM.*

##### Contemporary management

Current management was recently reviewed, revised and published in full [73]. Compared with the non-pregnant target of <600, European PKU pregnancy guidelines advocate a phenylalanine level of <360 micromol/L (or < 250 in many countries). This is based on the biochemical threshold for foetal vulnerability [74], given active placental phenylalanine transport [16].

Reproductive-age women with PKU or HPA, once identified, need referral to a specialist metabolic centre and/or a metabolic dietician. Pregnancies should be planned, and diet started preconception [73, 74].

Phenylalanine control generally improves once nausea settles, second trimester protein tolerance increases with maternal and foetal anabolism, and third trimester foetal uptake of phenylalanine parallels accelerated growth, lowering maternal levels [84]. With a PKU-affected foetus [84], (G Wilcox & D Green unpublished observations), maternal catabolism with impaired foetal phenylalanine uptake likely explains worsened phenylalanine control after 30 weeks’ gestation.

Catabolism from intercurrent infections or antenatal corticosteroids and/or psycho-social issues hindering dietary compliance may worsen phenylalanine control. BH4 may complement existing management, if accessible, and the mother responsive [74, 78, 79].

Post-partum resumption of usual diet is commonplace. Neuropsychiatric symptoms coincident with accelerated catabolism post-partum and high protein intake were reported anecdotally (personal communication Ian Chapman). Formal studies of post-natal depression in PKU are awaited. Breastfeeding is not contraindicated [73].

##### Homocystinuria

Genetic causes of hyper homocysteinaemia include cystathione beta synthase (CBS) deficiency or ‘classical homocystinuria’ (variably or not pyridoxine responsive) [85, 86], methylene-tetra hydro-folate reductase (MTHFR) deficiency [87] and cobalamin C (Cbl C) disease which results in combined methyl malonic acidaemia and homocystinuria due to deficient adenosyl-cobalamin [88]. These conditions may differ in clinical features and severity [86–88] but all are associated with elevated homocysteine levels and increased thrombosis risk.

Management includes co-factor replacement where relevant (e.g. B6 for B6 responsive CBS deficiency [86], folate for MTHFR deficiency [87] and B12 for Cbl C disease [88]), betaine to enhance remethylation to methionine, and protein restriction if appropriate with provision of methionine–free amino acid supplementation [86].

The impact and management of these conditions in pregnancy has been reviewed previously [5, 86, 89]; the main complications are maternal venous thromboembolism during pregnancy and post-partum [90]. MTHFR polymorphisms A1298C [91] and C677T which are relatively mild and common in the general population have been variably associated with recurrent miscarriage, [92–96] and reduced in-vitro fertilization success [97].

These conditions are therefore managed as a form of thromobophilia, with anticoagulation from the either the third trimester of pregnancy until 6 weeks post-partum or throughout pregnancy and the puerperium, depending on local protocol [5]. Since protein requirements increase during pregnancy, protein restrictions in place need adjustment to meet nutritional requirements.

##### Maple syrup urine disease

Maple syrup urine disease (MSUD) is an inborn error of branched chain amino acid (BCAA) metabolism due to deficiency of the intra-mitochondrial thiamine-dependent enzyme complex, Branched Chain Keto Acid Decarboxylase. This converts the BCAA derived keto-acids into their respective Coenzyme A derivatives for subsequent mitochondrial energy production [98–100].

Decompensation may occur with catabolic stress and/or excess protein intake, manifesting elevated BCAA, allo-isoleucine, and their respective keto-acids [101]. Of the BCAA, leucine is particularly neurotoxic [102], with isoleucine and valine significantly attenuating this [102]. The urine may have a characteristic ‘maple syrup’ odour, attributed to isoleucine-derived sotolone [103]. Clinical severity varies depending on the degree and type of enzyme deficiency. The most severe forms present neonatally, before newborn screening results return. Milder cases including ‘intermittent’ MSUD may not be diagnosed until adulthood. Newborn screening (NBS) programs should improve this outcome, long-term [101]. However, most women currently of childbearing age may not have been screened neonatally for MSUD. Presentations may vary according to age of symptom onset. Ketosis with raised BCAA, nausea, vomiting and progressive encephalopathy are characteristic: irritability, ataxia, hyponatraemia, brain oedema, coma and death may occur if untreated [104].

Management is by dietary restriction of natural protein, supplemented with BCAA-free fortified amino acid and valine supplements with strict monitoring of BCAA levels. Milder forms of MSUD may be thiamine responsive [99]. Long term suboptimal outcomes may be manifest by varying degrees of intellectual impairment and/or executive dysfunction [104].

In pregnancy, the risk periods are in the first trimester, where nausea and vomiting may result in catabolism from inadequate calorie intake, and post-partum when uterine involution increases the free amino acid pool. Intercurrent infections at any stage, and delivery by labour or caesarian section represent additional catabolic stressors.

Death with cerebral oedema has been reported 51 days post-partum, confounded by concurrent physical trauma [105], but successful pregnancies have been reported in the literature [106–109] as well as others (G Wilcox unpublished observations). Though rats, leucine-exposed neonatally, display long-term neuro-behavioural disturbance [110], no adverse outcomes are yet described in the children of mothers with MSUD, despite poor compliance and suboptimal metabolic control in some [108]. Leucine requirements increase disproportionately during late pregnancy [17], which may be protective. Clearly longer-term follow-up of such offspring is warranted.

#### Organic acidaemias

##### ‘Classical’ organic acidaemias

Of the organic acidaemias, isovaleric acidaemia (IVA) propionic acidaemia (PA) and methylmalonic acidaemia (MMA) are well described, intra-mitochondrial inborn errors of BCAA metabolism. Precursor amino acids are leucine for IVA, and valine, methionine, isoleucine and threonine, in addition to odd-chain-length fatty acids, for PA and MMA [104].

These conditions have typically presented neonatally or in infancy with acute metabolic decompensation associated with anorexia, nausea, vomiting, high anion gap metabolic acidosis and hyperammonaemia leading to coma if untreated [104]. Complications may appear in the post-acute recovery phase. Basal ganglia damage, neuropsychiatric symptoms, cardiomyopathy, pancreatitis, and more chronically, renal failure, may occur, especially in MMA [104, 111]. Many of these latter complications may represent mitochondrial dysfunction, since the toxic byproducts may accumulate intra-mitochondrially [112].

Survival has improved markedly in the last 40 years due to better diagnosis, treatment and reduced morbidity, especially with the implementation of expanded NBS programs [113]. However, most women currently of childbearing age have been born prior to expanded NBS.

Management is by natural protein restriction and supplementation with non-precursor amino acids. Carnitine is prescribed to enhance renal excretion of these short-chain fatty acids (isovaleric acid or propionic acid). Antibiotics may be prescribed to reduce gut bacterial production of propionic acid in PA & MMA. Some forms of MMA are vitamin B12 responsive and, with appropriate dosing, do not need significant dietary restriction [104, 114].

Successful pregnancies have been reported in IVA [115–118], PA [119, 120] and MMA (both B12 responsive and non-responsive forms) [121]. There are currently no studies to establish if subfertility is a problem or not.

Potential challenges include maintaining adequate caloric intake, especially in PA and MMA where anorexia is common even in the non-pregnant state [104], and avoidance of catabolism throughout pregnancy, labour, and the post-partum period, by providing adequate non-protein calories [4]. Titration of protein intake and carnitine dosage may be needed alongside close metabolic and nutritional monitoring [4, 114, 121].

*IVA:* There are four published case reports of isovaleric acidaemia in 8 pregnancies [115–118]. These were all successful and uncomplicated without post-partum decompensation. Plasma acyl carnitine monitoring showed variable decreases in gestational isovaleryl carnitine, reflecting decreased isovaleryl-CoA, from the second trimester as plasma leucine levels fell consistent with increased foetal anabolism, potentially conferring greater maternal metabolic stability [117, 118].

*PA:* There are currently 7 reported pregnancies in the literature [4, 106, 120], including 6 reviewed by Schwoerer et al. 2016 [119]. Four of these were uncomplicated and none experienced post-partum metabolic decompensation. Two pregnancies in one woman were significantly pre-term, with pre-eclampsia in both, and IUGR in one. All pregnancies were in known patients and managed pro-actively, continuing usual therapies and given IV glucose, ± I-carnitine, peri-partum in 6/7 cases. Mungan et al. reported uncomplicated C-section and lactation [120]. Unreported successful pregnancies have occurred [119]. Current reports are insufficent to preclude a pregnancy risk nor determine whether the pre-eclampsia, IUGR and pre-term births reported by Schwoerer et al. are isolated findings [119] or reflect PA-related placental mitochondrial stress.

*MMA:* Outcome in isolated MMA has been reviewed in a recent case series [121] (*n* = 17 with 13 completed pregnancies) including 9 additional cases from the literature; adding our own single case experience (Melbourne) totals 14 completed pregnancies. These reports encompass a spectrum of severity of MMA subtypes [4, 121–123], including one patient post renal transplant [124].

Complications reported include first trimester spontaneous abortions (3/18), increased Caesarean section rate (8/14 cases) for suspected foetal distress in 6/8, and renal function decline during pregnancy [125] in 2 cases, persisting post-partum in our case (GWilcox unpublished observations). One patient had metabolic decompensation at 24 weeks gestation and another mild hyperammonaemia [4]; in 4/13 birth weight was <2500 g. These previously diagnosed patients were maintained on usual therapies including carnitine and vitamin B12, where applicable and given IV glucose [121] or protein-free PN, peri-partum. No post-partum metabolic decompensations are reported, though our patient experienced transient unexplained dysphasia 5-6 weeks post-partum. Whether or not these obstetric complications relate to placental mitochondrial dysfunction in MMA warrants exploration. The long-term outcome of offspring from these pregnancies is to date unremarkable [121].

##### Combined MMA & homocystinuria

Cobalamin C disease an inborn error of cobalamin (vitamin B12) metabolism causing elevation in both methylmalonic acid and homocysteine, is generally responsive to high dose to parenteral hydroxycobalamin therapy ± oral betaine [88]. Despite optimal therapy, homocysteine remains elevated with attendant thromobosis, hence pregnancy, risk [88]. Case reports of successful pregnancy with maintenance of high-dose cobalamin therapy and appropriate thromboprophylaxis, e.g. aspirin, are reported [126, 127]. Of note, nitrous oxide anaesthesia should be avoided given its toxic effect on B12 metabolism [88].

##### ‘Cerebral’ organic acidaemias

This group of disorders are principally manifest by chronic neurological symptoms [111]. Glutaric aciduria Type 1(Ga1), is the best characterized. An intra-mitochondrial disorder of lysine and tryptophan metabolism, its presentation is varied. Febrile illnesses in early childhood may trigger encephalopathic crises resulting striatal damage and complex movement disorders [111]. NBS data has broadened the phenotypic spectrum and improved outcome [128]. Three case reports of 4 well-managed pregnancies, 2 delivered by caesarean section, are reported [4, 129, 130]. Protein restriction, avoidance of catabolic stress peripartum by intravenous glucose ± lipid, continuation of carnitine therapy (parenterally if needed) with careful biochemical monitoring likely supported a favourable outcome. Screening programs have identified asymptomatic women with apparently unremarkable pregnancy histories [130]. Three further untreated pregnancies in two women are reported. Neonatal carnitine deficiency lead to maternal GA1 diagnosis in one [130]. MRI changes with Sylvian enlargement in two offspring at 4 months’ age and bilateral temporal arachnoid cysts in one are described. Development at 3 and 5 years was reportedly normal [131]; clearly longer-term follow-up is needed.

#### Inherited disorders of carbohydrate metabolism

Disorders of carbohydrate metabolism encompass the glycogen storage diseases (GSD), disorders of fructose metabolism, galactosaemia and congenital disorders of glycosylation (CDG) [32].

##### Glycogen storage diseases (GSD)

There are 20 described GSD differentially affecting liver, muscle, heart and brain [132]. The hepatic (GSD 0, Ia, Ib, III, VI, IX & XI), muscle (GSD II, V, VII & X-XIV) and the muscle lysosomal GSD II, are amongst the better characterized. GSD III, affecting both liver and muscle, may be associated with cardiomyopathy [133].

The *hepatic GSDs* present with varying degrees of hypoglycaemia; chronic futile glycogenolysis and/or gluconeogenesis may cause deranged intermediary metabolism and end-organ damage. Frequent cornstarch and/or overnight tube feeding has enabled survival into adulthood in the more severe forms. Longer-term complications have emerged from the underlying conditions and their treatment. [133] Obesity, insulin resistance and progression to type 2 diabetes are increasingly seen in hepatic GSD [134].

Pregnancy is problematic in hepatic GSD due to increased glucose demands and energy requirements, especially in GSD1, where glycaemia is totally dependent on exogenous glucose [135–137]. Published reviews and case series report successful outcome with close glucose monitoring and increased cornstarch and/or overnight feeding to maintain euglycaemia [138, 139].

Maternal complications reported in *GSD1a* (glucose-6-phosphatase deficiency) include increased frequency/severity of hypoglycaemia, renal function decline, peripartum lactic acidosis, worsened hypertriglyceridaemia and foetal IUGR, macrosomia and neonatal hypoglycaemia. Maternal hepatic adenomata did not increase in this case series. [135]. Foetal death in utero at 33 weeks has been reported [140]. Late presentation and poor metabolic control with severe proteinuria, hypertension, IUGR and foetal distress resulted in emergency C-section of 412 g infant at 26 weeks and neonatal death 2 days later [141]. Progression of hepatic adenomata has been reported in GSD1b [138].

Survival in *GSD 1b* (glucose-6-phosphatase translocase deficiency), associated with neutrophil dysfunction, has followed availability of GCSF. Pregnancy outcomes have been reviewed [136] with intercurrent infections in all 5 pregnancies, progressive increases in glucose requirement towards term, maternal albuminuria in 3/5 and respiratory distress in 3/5 term infants.

*GSD III (glycogen debranching enzyme deficiency*) pregnancy outcome review [142] reports successful pregnancies, though cardiomyopathy worsened in 1 of 3 affected women and 4/15 infants had very low birth weight including one with persistent neurodevelopmental problems, despite expert management. Gluconeogenesis is intact in GSDIII [133]. How this impacts on already increased protein requirements late gestation [8] has not yet been studied.

Isolated *muscle GSD* such as McArdle’s (GSD V, myophosphorylase deficiency) in pregnancy have had limited review. The first full report of successful pregnancy and delivery in McArdle’s, reported in 1973, was essentially uncomplicated with minimal intervention [143]. Further uncomplicated C-section deliveries [144] with IV glucose [145, 146], are reported; C-section plus calf compression, without IV glucose, was complicated by compartment syndrome post-partum [147]. Retrospective review of 21 pregnancies by Findlay et al., reports minimal intervention and improved symptoms during pregnancy. Late gestational increased maternal lipid catabolism may be protective [143].

##### Galactosaemia

Of the galactosaemias ‘classical galactosaemia’ (due to galactose-1-phosphate pagesuridyltransferase deficiency or GALT deficiency) is the most common and managed by dietary restriction of lactose and galactose. This is associated with ovarian failure which may be primary or cause early secondary ovarian failure [148–150]. The exact mechanisms remain unclear but possibly occur in intrauterine life; secondary disturbances in glycosylation may occur [151]. Successful spontaneous pregnancies have occurred, even with absent AMH [152], and apparently without complications [153].

##### Disorders of fructose metabolism

Disorders of fructose metabolism include *hereditary fructose intolerance (HFI)* which may also be associated with secondary disturbance on glycosylation. One case report postulates maternal heterozygosity and gestational fructose ingestion leading to congenital abnormalities in a HFI affected foetus [154]. HFI symptoms, seen in homozygotes for the Aldolase B mutation, are generally prevented by complete dietary fructose exclusion. Pregnancies with HFI are reported and, with fructose aversion, unremarkable [155].

*Fructose 1,6 bisphosphatase deficiency* is a disorder of gluconeogenesis manifest by hypoglycaemia and lactic acidosis, triggered by fructose ingestion or fasting. Krishnamurthy et al. reported 3 completed pregnancies; one patient subsequently developed sensorineural hearing loss and early cognitive impairment [156].

*Congenital disorders of glycosylation* are frequently associated with reduced fertility possibly due to altered glycosylation of peptide hormones [157]. ‘Mirror syndrome’ with maternal oedema in the setting of hydrops foetalis has been reported in two pregnancies where the foetus was later diagnosed with CDG 1a [158]. This represents an example of a foetal IEM affecting the mother.

#### Inherited disorders of cholesterol metabolism

*Smith Lemli Opitz syndrome*, an IEM of cholesterol synthesis, causes 7 & 8 dehydro-cholesterol accumulation, Sonic Hedgehog signaling pathway disruption with characteristic dysmorphic features, autistic spectrum disorder and variable intellectual disability. Treatments, with limited evidence-base include cholesterol and/or statin therapy. Successful pregnancy reported in a mild case, was managed with dietary cholesterol loading from 29 weeks [159].

##### Abetalipoproteinaemia

This LDL synthesis disorder severely impairs absorption, transport and delivery of cholesterol and fat-soluble vitamins, especially Vitamin E. Treatment requires administration of high-dose fat-soluble vitamins. Adverse pregnancy outcomes are reported with poor compliance to fat soluble vitamin replacement [160–162]. Conversely successful outcome has been reported with adequate vitamin replacement and monitoring [163]. Vitamin A replacement should be continued to maintain adequate levels, as deficiency, as well as excess, can result in adverse foetal outcome [162].

### Inborn errors of mitochondrial energy metabolism

Substantially increasing energy requirements during pregnancy, particularly for mitochondrial-rich organs such as heart, kidneys, liver and placenta, renders energy supply pivotal. Mitochondrial energy metabolism may be affected by disorders of ketogenesis and ketolysis, mitochondrial fatty acid oxidation, and disorders of the respiratory chain including those resulting from maternally-inherited mitochondrial DNA mutations [32].

#### Disorders of ketogenesis & ketolysis

Variable pregnancy outcomes have been reported in this group [164]. Early gestational fasting ketone availability and utilization is critical, especially if nausea and vomiting limits intake.

In *HMG CoA Lyase deficiency*, prone to hypoketotic hypoglycaemia and metabolic acidosis, early foetal losses and maternal death at 9 weeks gestation were reported [4]. A successful pregnancy was managed with L-carnitine, protein-intake adjustment and glucose calories [123]. Despite IV 10% glucose and IV L-carnitine, decompensation occurred during labour, necessitating emergency C-section [123]. Two further successful pregnancies recently reported, were managed by oral glucose, carnitine and overnight cornstarch during pregnancy, with planned C-Section and total parenteral nutrition peri- and post-partum providing >2000 Kcal per day. This provided sufficient calories in the face of potent catabolic stress and increased energy requirements [165].

*Defects of ketolysis* can result in excessive ketosis. Emergency C-section at term for foetal bradycardia was reported in a woman with *beta-ketothiolase deficiency* [166]. These disorders, and pregnancy considerations, are reviewed [164].

#### Disorders of mitochondrial fatty acid oxidation

These disorders exemplify how a foetal IEM may impact on the mother, and the insights a rare condition may bring to the wider field.

The association of maternal Acute Fatty Liver of Pregnancy (AFLP) and/or Haemolysis, elevated liver enzymes, low platelets (HELLP) syndrome with foetal Long Chain Hydroxy Acyl CoA Dehydrogenase deficiency (LCHAD) first noted in 1991, has been extensively studied [21, 167] and recently reviewed [29].

**Fig. 4:**
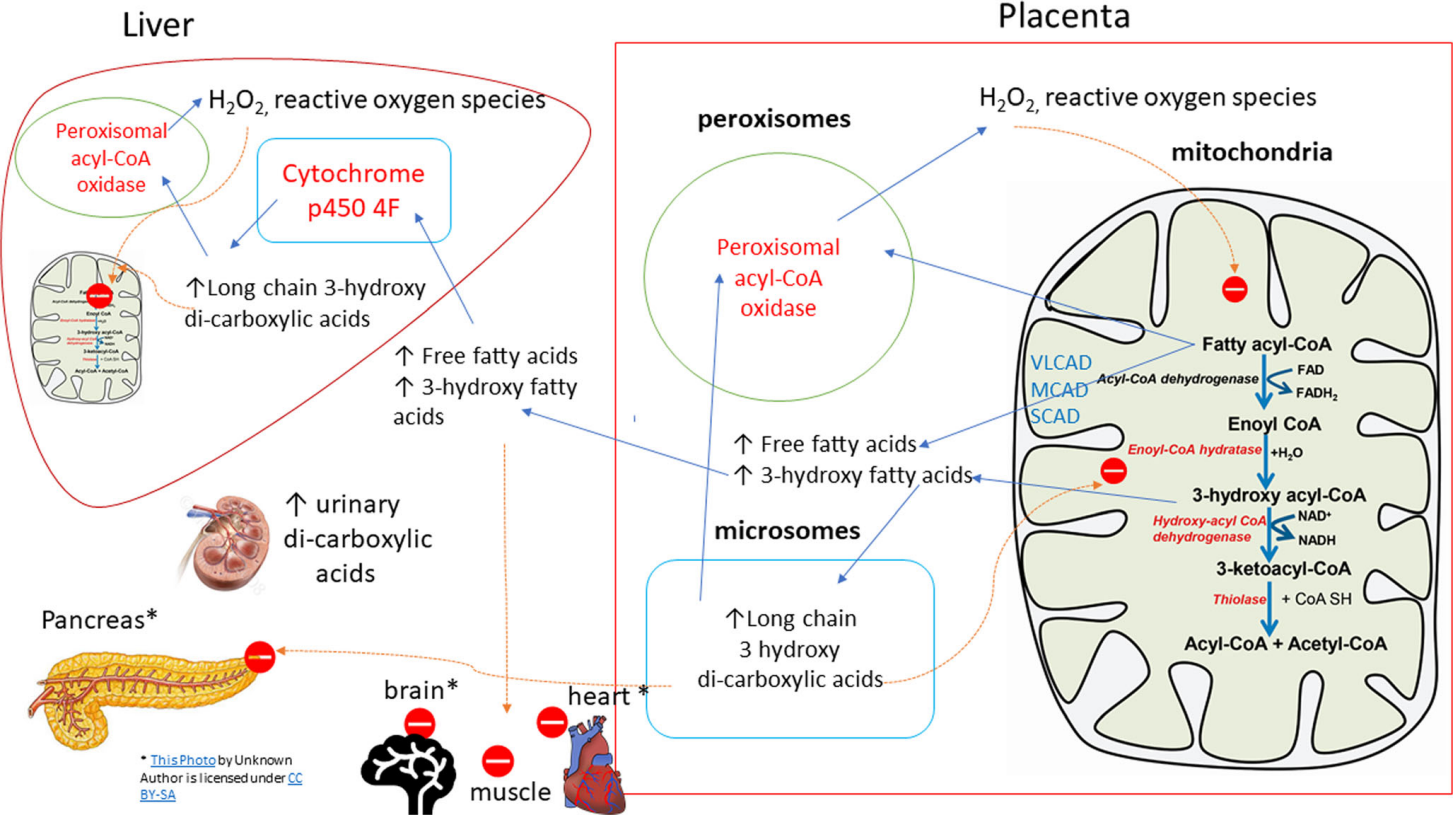
**Mitochondrial fatty acid β oxidation pathway & interplay between foeto-placental and maternal metabolism in LCHAD.** Classical β-oxidation pathway involves: dehydrogenase by acyl-CoA dehydrogenase and hydration, dehydrogenation and thiolyic cleavage is catalyzed by the -mitochondrial trifunctional protein (MTP, highlighted in red color). MTP consists of: enoyl-CoA hydratase,hydroxy acyl-CoA dehydrogenase & thiolase activity. The straight arrows represent products and bent arrows represent the involvement of co-factor in this enzyme-catalyzed reaction. Fetal long chain 3-hydroxy acyl-CoA dehydrogenase (LCHAD) deficiency results in accumulation of 3-hydroxy fatty acids in the placenta, since the fetal part of placenta is identical to the genetic makeup of the fetus. Increased accumulation of placental free fatty acids and 3-hydroxy fatty acyl-CoA cause oxidative stress, mitochondrial dysfunction and placental lipotoxicity. Further, lipolysis induced in the third trimester of pregnancy would also trigger the accumulation of fatty acid intermediates, which are shunted from the placenta to the maternal circulation, where they can promote oxidative and nitrosative stress. These fatty acid intermediates reach the maternal liver resulting in microvesicular steatosis, hepatic mitochondrial dysfunction and hepatocyte lipoapoptosis. Source: Adapted from Natarajan & Ibdah (reference [29]) Int J Mol Sci. 2018 Jan; 19(1): 322. Published online 2018 Jan 22. doi: 10.3390/ijms19010322

*Foetal homozygosity for the LCHAD enzyme includes the placenta*. Incomplete mitochondrial beta-oxidation leaves residual substrate being catalyzed by alternative pathways in peroxisomes and microsomes. Peroxisomal, unlike mitochondrial, beta-oxidation, does not yield ATP. Instead, by-products of blocked pathways yield peroxide radicals and other pro-oxidant species, further compromising placental mitochondrial function. Since foetal and maternal placental circulations, are juxtaposed, toxic by-products freely enter the maternal circulation where effects on foetal sub-cellular metabolism are mirrored in maternal organs [21, 29] Fig. 4. The maternal LCHAD heterozygote may be more vulnerable to mitochondrial dysfunction, including beta-oxidation [29]. Compromised hepatic mitochondrial function likely underlies acute fatty liver of pregnancy (AFLP) [168]. Other maternal organs may be affected. Pancreatitis, renal dysfunction and cerebral effects may be seen [29]. Pathophysiological derangements encompassing the clinical spectrum of AFLP and HELLP to pre-eclampsia are reported [29]. Other foetal [169–172] and maternal [169, 173] FAOD are reported with these syndromes.

Accumulating evidence suggests placental mitochondrial dysfunction may in fact underlie many pathological conditions in pregnancy [12, 100, 174–179] (Tables 2 & 3*).*

**Table 2:** Mitochondrial dysfunction in IEM with implications for pregnancy

Primary disorders of mitochondrial energy metabolism	Mitochondrial dysfunction secondary to IEM
Mitochondrial DNA^a^	
*Respiratory chain disorders:*	*Urea Cycle Disorders* ^*c*^ *:*
MELAS	OTC deficiency
Chronic Progressive External Ophthalmoplegia	CPS deficiency
	Citrullinaemia
Autosomal	*Organic acidaemias* ^*d*^ *:*
*Mitochondrial FAOD* ^*b*^ *:*	Isovaleric acidaemia
SCAD	
MCAD	Propionic acidaemia
VLCAD	Methyl malonic acidaemia
LCHAD	
CPT1	*Cerebral organic acidaemias*^*d*^:
CPT2	
MADD^e^	Glutaric aciduria Type 1

References ^a^ [180] ^b^ [21], ^c^ [38, 111, 112]

^e^Creanza A JIMD Rep. 2017 Jul 7. doi: 10.1007/8904_2017_38

*MELAS* Mitochondrial encephalomyopathy, lactic acidosis, and stroke-like episodes; *FAOD* Fatty acid oxidation disorders; *SCAD* Short**-**chain acyl-CoA dehydrogenase deficiency; *MCAD* Medium-chain acyl-CoA dehydrogenase deficiency; *VLCAD* Very long-chain acyl-CoA dehydrogenase deficiency; *LCHAD* Long-chain 3-hydroxyacyl-CoA dehydrogenase deficiency; *CPT1* Carnitine palmitoyltransferase I deficiency; *CPT2* Carnitine palmitoyltransferase II deficiency; *MADD* Multiple Acyl-CoA Dehydrogenase Deficiency or Glutaric aciduria Type 2

**Table 3:** Acquired causes of mitochondrial dysfunction which may impact on pregnancy

Acquired mitochondrial dysfunction	Potential mechanism(s)
Viruses	
Influenza A^a^ Influenza B^b^ Hepatitis C virus^c^ Human Immunodeficiency Virus (HIV)^c^	Inflammatory cytokines ↓ mitochondrial fatty acid oxidation enzyme activity Oxidative stress and Δ mtDNA mtDNA depletion & apoptosis
Nutrient deficiencies^d^	
Hypoxia & ischaemia	↓cellular respiration
↓phosphate	↓ATP production
↓ magnesium	
Thiamine deficiency	↓Pyruvate Dehydrogenase function
Riboflavin deficiency	↓Respiratory chain function
Niacin deficiency	
Carnitine deficiency	↓β oxidation of long chain fatty acids
Endocrine disorders	
Hypothyroidism^e^	↓β oxidation of long chain fatty acids via ↓carnitine
Drugs of abuse	
Tobacco smoking^c^	CO ↓cellular respiration respiratory chain complex III ↓
Ethanol^c^	Altered NADH/NAD+ redox state
Therapeutic drugs	
Anaesthetic agents: propafol^c^	Inhibits complex 1 of the respiratory chain

*mtDNA* mitochondrial DNA; NADH/NAD+ = nicotinamide adenine dinucleotide hydride in reduced/oxidized states; *ATP* Adenosine triphosphate

*Sources:* ^a^ [175], ^b^ [176], ^c^ [179], ^d^ [100]

Recently, *maternal* LCHAD, with foetal heterozygosity, was reported. Pregnancy was initially uncomplicated. At 32 weeks’ gestation, maternal tachycardia, raised CK and lactate responded temporarily to increased medium chain triglyceride (MCT) oil dosage. Recurrent tachycardia determined C-section at 34 weeks. Acylcarnitine profile, improved in early pregnancy, had deteriorated, likely reflecting late gestational accelerated lipolysis [181].

Interestingly *maternal VLCAD* may stabilize with an unaffected foetus demonstrating the quantitative importance of placental beta oxidation of long chain fatty acids [23, 25, 169, 182].

*Systemic carnitine deficiency* in pregnancy has been associated with decreased stamina and cardiac arrhythmia [183].

### Organelle-based disorders

#### Primary mitochondrial disorders

Pregnancy complications are reported in mitochondrial IEM. Most publications relate to MELAS. A retrospective case series by De Laat [180] reports premature delivery rates of 23.5% (5/23 < 32 weeks), pre-eclampsia 12% and gestational diabetes mellitus 11%. Other case reports include neuromuscular deterioration and lactic acidosis [184], status epilepticus [185], pulmonary oedema [186], pre-eclampsia [187, 188] with magnesium sulphate infusion sensitivity [189] and post-partum pulmonary embolism [190]. These complications likely represent precarious placental function in mitochondrial disease and gestational energetic strain on vital maternal organs.

#### Peroxisomal disorders

These include Refsum’s disease, a disorder of branched-chain fatty acid oxidation, with both infantile and adult-onset forms. Chloroplast-derived phytanic acid, from ruminant fat, cannot be metabolized, accumulating in the nervous system affecting hearing, retinal function, peripheral nerves. Phytanic acid levels are managed by diet-restriction ± plasmapheresis and avoidance of lipolysis, which can release phytanic acid from adipose tissue triggering neurological decompensation. A childhood-onset Refsum’s disease pregnancy, reporting reduced phytanic acid control late gestation, despite close dietetic management [191], likely reflected accelerated lipolysis [12, 14].

#### Lysosomal storage disorders

This large group of IEM includes Gaucher’s disease, Fabry’s Disease, Pompe Disease (GSD II- a lysosomal muscle GSD) as well as the Mucopolysaccharidoses (MPS) and related disorders [32]. There are, currently, recombinant lysosomal enzyme replacement therapies (ERT) for many of these conditions as well as emerging treatments such as substrate reduction, chaperone and gene therapies [192]. Successful pregnancies are reported, including for Gaucher [193, 194], Fabry [195], Pompe [196] and MPS disorders [197–199]. ERTs do not cross the placenta and have been given in pregnancy without complications [198–200]. Disease-specific registries exist, from which basic pregnancy data could be derived. Pompe disease has been associated with transiently worsening muscle weakness late gestation [201]. Patients with MPS now reach adulthood in increasing numbers; the pregnancy experience in this group was recently reviewed [197].

## Conclusions

This review is not exhaustive and there remain significant gaps in the literature, with many pregnancies not recorded; some areas reviewed elsewhere are not covered here and others where information is extremely limited. There is an urgent need for systematic data collection with an international registry of all pregnancies, and follow-up of all offspring, associated with maternal IEM. Long-term effects of either the primary metabolic disorder or the nutritional and/or pharmacological management warrant investigation. Long-term benefits of expanded newborn screening programs should be examined. Undiagnosed IEM may need consideration where there is hyperemesis gravidarum, liver failure or neuropsychiatric disturbance. The convergence of understanding between complications of various IEM in pregnancy, as well as acquired disorders, is intriguing. Advancing the pathophysiology and management of these rare IEM may illuminate mechanisms underlying more common conditions such as pre-eclampsia, gestational diabetes, and intra uterine growth retardation.

## Footnotes

1 Goldstein R, Smith N, Strauss BJG & Wilcox G. “Protein aversion and disordered eating in OTC deficiency: a challenge for pregnancy and post-partum management in a female heterozygote” 25th DMIMD, London, UK April 2011
